# The Efficiency of In Vitro Differentiation of Primate iPSCs into Cardiomyocytes Depending on Their Cell Seeding Density and Cell Line Specificity

**DOI:** 10.3390/ijms25158449

**Published:** 2024-08-02

**Authors:** Yuliia Tereshchenko, Stoyan G. Petkov, Rüdiger Behr

**Affiliations:** 1 Research Platform Stem Cell Biology and Regeneration, German Primate Center–Leibniz Institute for Primate Research, Kellnerweg 4, 37077 Göttingen, Germany; ytereshchenko@dpz.eu (Y.T.); spetkov@dpz.eu (S.G.P.); 2 DZHK (German Centre for Cardiovascular Research), Partner Site Lower Saxony, 37077 Göttingen, Germany

**Keywords:** iPSCs, cardiomyocyte, in vitro differentiation, cell seeding density, human, rhesus monkey, non-human primate

## Abstract

A thorough characterization of induced pluripotent stem cells (iPSCs) used with in vitro models or therapeutics is essential. Even iPSCs derived from a single donor can exhibit variability within and between cell lines, which can lead to heterogeneity in results and hinder the promising future of cell replacement therapies. In this study, the cell seeding density of human and rhesus monkey iPSCs was tested to maximize the cell line-specific yield of the generated cardiomyocytes. We found that, despite using the same iPSC generation and differentiation protocols, the cell seeding density for the cell line-specific best differentiation efficiency could differ by a factor of four for the four cell lines used here. In addition, the cell lines showed differences in the range of cell seeding densities that they could tolerate without the severe loss of differentiation efficiency. Overall, our data show that the cell seeding density is a critical parameter for the differentiation inefficiency of primate iPSCs to cardiomyocytes and that iPSCs generated with the same episomal approach still exhibit considerable heterogeneity. Therefore, individual characterization of iPSC lines is required, and functional comparability with in vivo processes must be ensured to warrant the translatability of in vitro research with iPSCs.

## 1. Introduction

The development of a mammalian organism is a complex process that is still only partially understood. Temporally and spatially ordered information defines the expansion of numerous cell types in the correct number [1], orientation and size [2]. To form a functioning organ, the cellular composition of the organ-forming tissue is important; each organ, therefore, consists of specialized cell types whose distribution is defined within narrow limits in terms of their number and cell size [2]. In the heart, there are four main cell types: cardiac muscle cells, fibroblasts, endothelial cells and pericytes. While endothelial cells and fibroblasts together make up about two-thirds of the cells in the heart, their contribution to the biomass is less than 10%. Cardiac muscle cells, on the other hand, make up about 10% of the cells in the heart, but they comprise about two-thirds of the biomass. These figures show that the density of the cells of different cell types in a 3D context in vivo is important for the normal development of tissues and organs.

Embryonic stem cells (ESCs) and induced pluripotent stem cells (iPSCs) are functionally comparable pluripotent stem cells (PSCs) that can form whole organisms, as demonstrated in tetraploid complementation experiments [3,4]. PSCs have great potential regarding both cell replacement therapy [5,6,7,8,9] and the in vitro testing of substances [10,11,12]. Based on these functions, PSC differentiation assays could be established in toxicology as a replacement method for animal experiments [12,13].

The establishment of well-characterized cell lines in combination with well-defined and validated protocols is necessary for optimized cell replacement therapies as well as for standardized in vitro methods to replace animal experiments in drug testing (pharmacology and toxicology). However, the generation of iPSC lines is subject to multiple variables, both methodological (e.g., the type of reprogramming, the cell culture medium and the adherence matrix) and biological (e.g., the cell type being reprogrammed and the age of cells), and cell heterogeneity appears both within a cell line [14,15,16] and between cell lines [14,15].

In the present study, we used two human and two rhesus monkey (*Macaca mulatta*) fibroblast-derived iPSC lines generated in our laboratory using the same method [17] and differentiated them following a protocol established and standardized for both species [17]. The project aimed to determine an optimized starting cell density for iPSCs applicable to all iPSC lines for a reproducible, high-efficiency process of differentiation into cardiomyocytes.

## 2. Results

**Figure 1:**
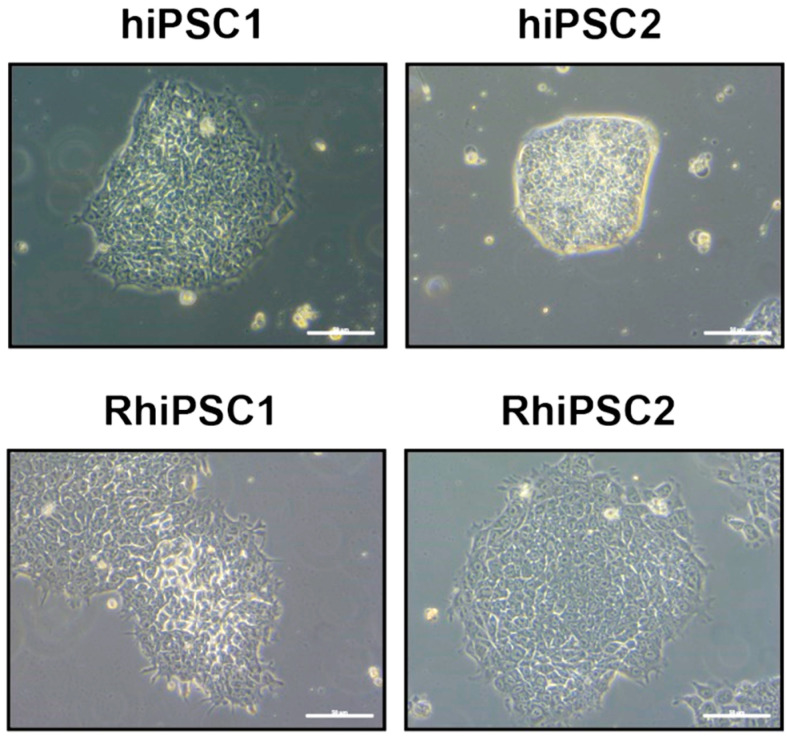
The morphology of human (hiPSC1 and hiPSC2)- and rhesus macaque (RhiPSC1 and RhiPSC2)-induced pluripotent stem cell colonies in feeder-free culture conditions. Scale bars: 50 µm.

Two human (male hiPSC1 and female hiPSC2) and two rhesus macaque (male RhiPSC1 and female RhiPSC2) iPS cell lines were used to generate cardiomyocytes in vitro. The iPSCs exhibited the typical features of pluripotent stem cell colonies, such as well-defined borders and a high nucleus/cytoplasm volume ratio. The rhesus iPSC colonies were generally less compact and showed less defined colony borders (Figure 1). However, their ability to differentiate into derivatives of the three embryonic germ layers was demonstrated previously in embryoid body formation and in a teratoma formation assay [17].

**Figure 2:**
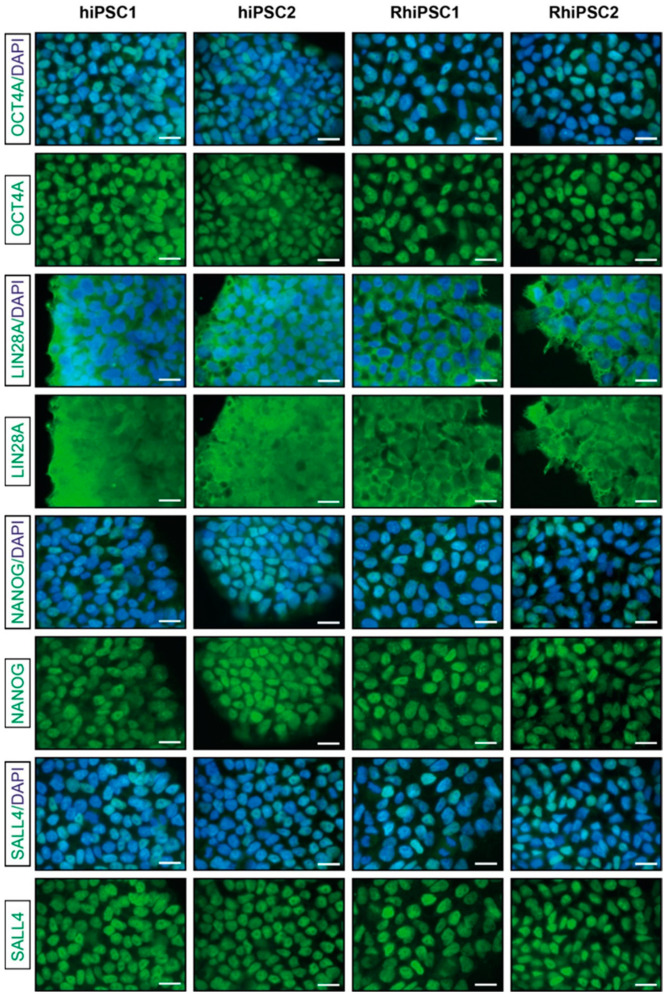
Immunofluorescence characterization of hiPSCs and RhiPSCs determined via pluripotency marker detection at the time the experiments reported in this study were conducted. A general characterization of the cell lines was completed earlier [17]. All four cell lines exhibited robust immunoreactivity for the key pluripotency markers OCT4A, NANOG, SALL4 and LIN28. The cell nuclei were stained with DAPI. Scale bars: 20 µm.

Prior to the actual study, the expression of the selected pluripotency markers of all four iPSC cell lines was re-analyzed to confirm the pluripotent state of the cells [17]. Figure 2 shows representative pictures of pluripotency marker expression for all four iPSC lines of the chosen passages from the working cell bank. A marker analysis confirmed the continuous expression of key pluripotency factors, namely the transcription factors NANOG, OCT4A (POU5F1) and SALL4 and the cytoplasmic RNA-binding protein LIN28A.

**Figure 3:**
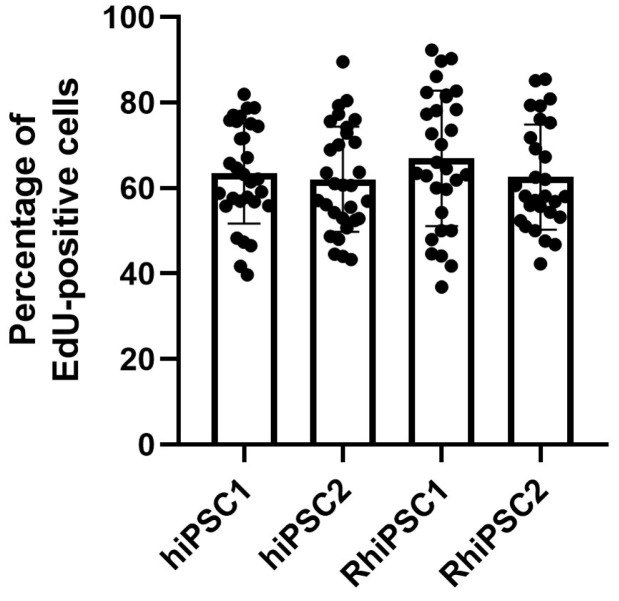
Percentage of EdU-positive cells. The cell lines hiPSC1, hiPSC2 and RhiPSC2 show an average EdU incorporation rate of around 60%. The average incorporation rate of RhiPSC1 is slightly higher.

To further characterize the iPSC lines, we analyzed their relative proliferative activity using EdU incorporation via a click chemistry reaction. One day after cell splitting, EdU was added to the cells for two hours. All the cell lines showed an EdU incorporation rate of about 60% or slightly higher (hiPSC1 63.48 ± 11.76%, hiPSC2 62.08 ± 12.33%, RhiPSC1 66.94 ± 15.84% and RhiPSC2 62.6 ± 12.28%) (Figure 3, Table S1). In general, the average proliferative activity of all four cell lines used in this study was similar.

In recent years, several protocols for myocardial differentiation have been published that are primarily tailored to human cell lines [18]. However, the translatability of these approaches to cells of other primate species is not guaranteed. Initially, growth factor-free cardiac differentiation protocols for human cells did not work for NHP iPSCs in our setting [14]. Therefore, we optimized a protocol for the cardiomyocyte differentiation of human iPSCs that was also suitable for non-human primate iPSCs. Finally, a hybrid differentiation method including small molecules (CHIR99021) and growth factors (activin A and BMP4) was successful and resulted in cardiomyocyte differentiation from NHP iPSCs. At the same time, this modified protocol still allowed for human iPSCs’ differentiation into cardiomyocytes [17]. When using this protocol, cardiac differentiation starts when the iPS cell confluency reaches 80–90%. However, despite the establishment of the common primate iPSC differentiation protocol, early cardiac differentiation experiments involving several cell lines showed considerable variability in cardiomyocyte yield across batches of experiments. This was evidenced by the number of beating cardiomyocyte clusters that occurred in the course of the protocol and the percentage of cardiac troponin T (cTNT, encoded by the *TNNT2* gene)-positive cells 12 days after the onset of differentiation (Y.T., unpublished). In contrast to human cells, which regularly showed high cardiomyocyte differentiation rates (>85%), rhesus macaque cells differentiated less efficiently and often showed less than 50% or sometimes even no cardiomyocytes after the established protocol. To reduce the influence of inter-experiment variability, a working iPSC bank was created to prevent run-to-run variations as confounding factors in the differentiation of iPSCs into cardiomyocytes.

**Figure 4:**
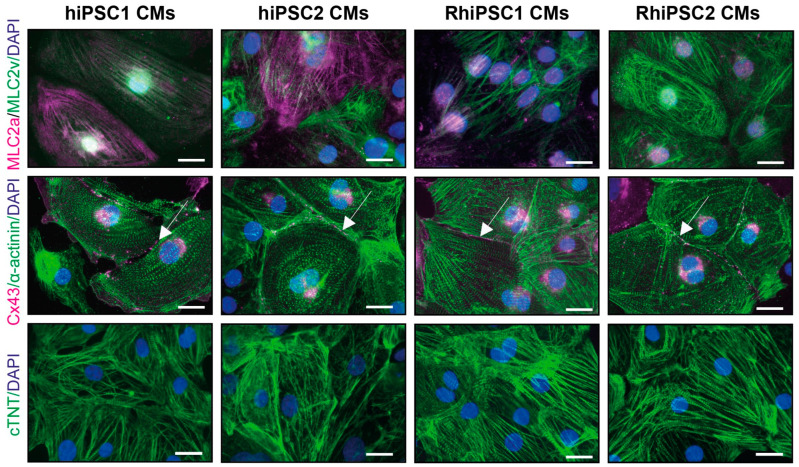
Marker expression of iPSC-derived cardiomyocytes. Immunostaining of hiPSC- and RhiPSC-derived cardiomyocytes. Cardiomyocyte markers, such as myosin light chain 2 ventricular (MLC2v), myosin light chain 2 atrial (MLC2a), α-actinin, cardiac troponin T (cTNT; encoded by the *TNNT2* gene) and connexin 43 (Cx43) were detected. The fluorescence images show the typical structure and morphology of iPSC-cardiomyocytes forming striated patterns of sarcomeres. The cell nuclei were stained with DAPI. Scale bars: 20 µm.

**Figure 5:**
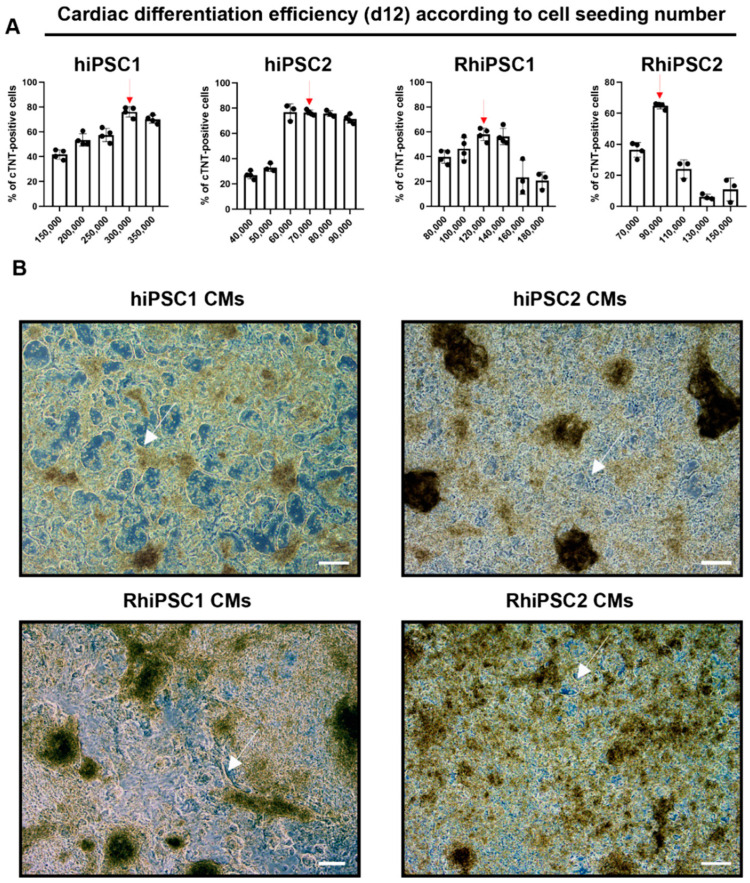
Directed cardiac differentiation of hiPSCs and RhiPSCs. (**A**) The flow cytometric measurement of cTNT (encoded by the *TNNT2* gene) signal shown at day 12 of differentiation in the four cell lines is seeded at different cell numbers/well. The red arrow indicates the number of cells/well resulting in the highest percentage of cTNT-positive cells for each of the four cell lines. (**B**) The morphology of human and rhesus iPSC-CMs is shown upon the optimization of the seeding number on day 12 from the beginning of differentiation. The white arrows indicate beating cardiac tissues that form net-like structures. Scale bars: 100 µm.

Using this working iPSC bank, we aimed to determine the optimal cell seeding density at the start of differentiation as the only variable in our experiments. All the cells that were cTNT-positive in the flow cytometric analysis were considered in the subsequent experiments to be cardiomyocytes (see Figure 4 for comparison). After a series of preliminary experiments to determine the broad range of possible cell seeding densities (data not shown), five to six different cell seeding densities (cells/well) were chosen for each cell line to obtain the highest percentage of cardiomyocytes (Figure 5; *n* ≥ 3 differentiations/cell line and density). For an exemplary overview of hiPSC1 culture morphology during differentiation, see Supplementary Figure S1. The upper limit of cell seeding density corresponded to 100% cell confluence at the beginning of cardiac differentiation.

Due to the different characteristics of the cell lines, the hiPSC1 line was tested in 50,000 cells/well increments, the hiPSC2 in 10,000 cells/well increments and the two RhiPSC lines in 20,000 cells/well increments. Ultimately, seeding 300,000 cells/well of hiPSC1 and 70,000 cells/well of hiPSC2 resulted in the highest percentages of cardiomyocytes generated: 76.19 ± 4.13% and 76.52 ± 1.99%, respectively (Figure 5A, Tables S2–S5). In contrast to human cells, the maximum differentiation efficiency of RhiPSC lines 1 and 2 remained below 70% and was 57.95 ± 5.1% at 120,000 cells/well and 64.82 ± 1.98% at 90,000 cells/well, respectively. It is noteworthy that in RhiPSC2, only one cell seeding density (90,000 cells/well) resulted in a differentiation efficiency of >60%, while the other seeding densities showed less than 40% differentiation efficiency.

Figure 5B shows bright field images illustrating the typical morphology of cardiac cells during the differentiation of iPSCs on d12. The development of reticular structures is a common feature of early cardiomyocyte cultures prior to the selection process. The beating of this network began on days 7–8 of differentiation.

**Figure 6:**
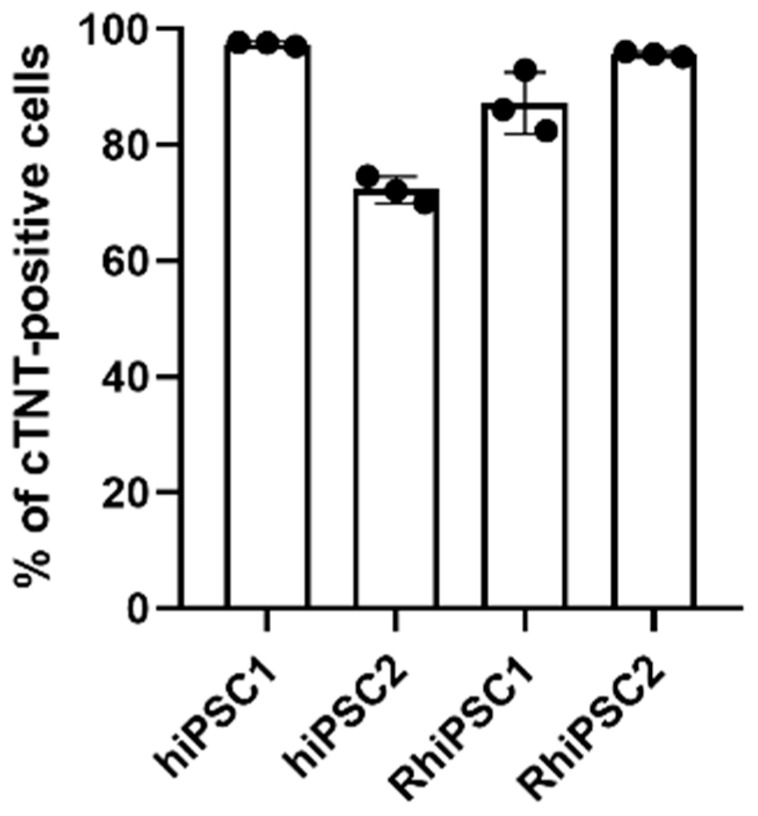
Flow cytometry analysis of the purity of the hiPSCs- and RhiPSC-derived cardiomyocyte populations after seven days of metabolic selection with lactate (as percentage of cTNT-positive cells; *n* = 3/cell line).

The first split of the cardiomyocytes was carried out on the basis of the results shown in Figure 5. Lactate was then used for the metabolic selection process of the cardiomyocytes, as only muscle cells can use lactate as a substitute for glucose. This led to a reduction in the non-cardiomyocyte cell population, resulting in 97.47 ± 0.37%, 72.26 ± 2.32%, 87.2 ± 5.35% and 95.68 ± 0.46% of cardiomyocytes in hiPSC1, hiPSC2, RhiPSC1 and RHiPSC2, respectively (Figure 6, Table S6). Thus, the cardiomyocyte selection in the rhesus cells also resulted in relatively pure populations of cardiomyocytes.

Following the second split of the cardiomyocytes after selection, the cells were analyzed for the presence of cardiac markers, as shown in Figure 4. Each of the four cell lines exhibited different cell morphologies within the cell line, including round, spindle-like and polygonal shapes. However, despite the different cell morphologies, immunofluorescent staining of the cells showed the expression of cardiac markers, such as myosin light chain 2 ventricular (MLC2v), myosin light chain 2 atrial (MLC2a), α-actinin and cardiac troponin T (Figure 4). In addition, these proteins formed organized sarcomere structures, indicating the development of cardiomyocytes. Cell–cell contacts relevant for the electrical coupling of cardiomyocytes were detected by staining for connexin 43 (Cx43; Figure 4). Cx43 is essential for proper electrical signal propagation and a synchronized heartbeat. Representative videos of beating cardiomyocytes derived from hiPSC1 and RhiPSC1 are shown in Supplementary Videos S1 and S2. Interestingly, RhiPSCs mainly expressed MLC2v, while in hiPSC lines, both MLC2v and MLC2a were present. To further corroborate the differentiation of cardiomyocytes, we also examined the expression of selected genes from the hiPSC1 line as undifferentiated stem cells and stem cells after differentiation (Figure S2). While the core pluripotency factors *POU5F1*, *NANOG* and *SOX*2, as well as other pluripotency genes, exhibited a high expression of the iPSCs, these genes were not expressed in the cardiomyocytes. Conversely, the cardiomyocytes exhibited a high expression of both characteristic transcription factors, such as *GATA4*, *GATA5*, *GATA6* and *TBX5*, as well as transcripts encoding structural proteins, such as *TTN*, *TNNT2* and *TNNC1*. In contrast, the cardiomyocyte markers were not expressed in iPSC. Together, these data demonstrate that cardiac muscle cells were indeed generated, but they also indicate nuanced differences in the expression of cardiac markers in the different cell lines.

## 3. Discussion

Induced PSCs have multiple applications, including regenerative medicine, disease modeling in the dish [11,12], drug testing [10,11,12] and developmental biology [12,19,20].

An essential basis for obtaining meaningful data in all these applications is the in vitro reproducibility of the in vivo developmental processes. While in vivo, the development of an organism follows relatively strict temporal and spatial rules, and the cell size and density within a particular tissue are also closely coordinated [2], it is challenging to model these developmental processes precisely in vitro using PSC lines. One reason for this is the heterogeneity of PSC lines. In the present study, we tested only one simple parameter: cell seeding density and its effect on cardiomyocyte differentiation in vitro using a standardized protocol validated for human and non-human primate cell lines [17]. In addition, all the cell lines were generated according to the same protocol [17]. Nevertheless, we found that the optimal cell seeding density for efficient cardiomyocyte differentiation varied greatly between the lines; between the two human cell lines, it varied by a factor of four. Moreover, the percentage of non-cardiomyocytes that survived metabolic selection with lactate instead of glucose as an energy source was significantly higher in hiPSC2 than in the other cell lines (Figure 6). However, the identity of the cTNT-negative cells that survived the lack of glucose is not known and requires further analyses. Nevertheless, the data shown in Figure 6 further demonstrate clear heterogeneity between the cell lines used in the present study. The reasons for our findings may include sex and species differences. However, other differences at the molecular level, such as epigenetic, genetic and transcriptomic differences between the lines, may also be responsible for our results. In fact, it is well known that there are differences between fibroblast populations, between organs, and even within an organ [21]. This also holds true for the skin [21]. The epigenetic state of the starting cell population in our study’s skin fibroblasts, in particular, could play a role in iPSC heterogeneity if epigenetic differences between individual cells reprogrammed to pluripotency remain during the reprogramming process [22]. Moreover, epigenetic marks common to all fibroblasts may be erased or modified with varying efficiency during reprogramming [23]. For an in-depth analysis of different iPSC lines, epigenetic and transcriptomic characterization is important. Since we have already seen large differences between the iPSC lines in our simple experimental design for the current study, it is very important to validate the cell lines against each other on the one hand and—probably even more importantly—to validate the processes modeled in vitro using PSCs against in vivo processes on the other hand. The lack of proper validation results in the concern that non-validated methods and cell lines can lead to misinterpretations and wrong—and in the worst cases, fatal—conclusions in the fields of PSC-based disease modeling, drug testing and development of regenerative therapies.

In summary, the in vitro data obtained through only one PSC line should be considered with caution, as one cell line may not be representative of other cell lines and, furthermore, may not be representative of the situation in vivo. Thus, on the one hand, we consider the use of several cell lines to be important in order to obtain reproducible and thus robust in vitro data. On the other hand, we consider in vivo validation of PSC-based in vitro assays to be urgently required. However, the variability between the different differentiations found under a given condition (such as cell line or seeding density) was low and demonstrated the high reproducibility of our results for individual cell lines within a given set-up.

## 4. Materials and Methods

This publication is mainly based on results obtained by Y.T. as part of her doctoral project. The relevant materials and methods have therefore already been described in Y.T.’s dissertation, which can be accessed at http://doi.org/10.53846/goediss-10407. Since parts of Y.T.’s dissertation and this publication are based on the same experiments, the description of the materials and methods is essentially very similar or even partially identical.

### 4.1. Cell Culture and Lines

Induced pluripotent stem cell lines (iPSCs; see Table 1) had been previously generated and kept in the Degenerative Diseases Platform stock at the German Primate Center (DPZ). Rhesus monkey RhiPSC1 and RhiPSC2 and human hiPSC1 and hiPSC2 cells were obtained by episomal reprogramming and maintained at 37 °C and 5% CO_2,_ as published previously [17]. Cells were sourced from the working stock of the same passage and used for no more than 10–20 passages. Cells were fed daily with a UPPS medium containing StemMACS™ IPS-Brew XF (Miltenyi Biotec, Bergisch Gladbach, Germany, #130–104-368), 0.5 μM Chir99021 (Stemgent, Cambridge, USA #04-0004) and 1 μM IWR-1 (Sigma-Aldrich, St. Louis, MA, USA #I0161) in feeder-free conditions on plates/wells coated with 0.17 mg/mL of Geltrex™ (Thermo Fisher Scientific, Waltham, MA, USA, #A1413202).

Passaging: Versene 1:5000 (1X; Gibco, Waltham, MA, USA, #15040-033) was used to passage the cells. The medium was removed and Versene was added for 2 min at RT, after which the reagent was removed and fresh Versene was added again for another round of 2 min at RT. Next, the cells were detached in UPPS supplemented with 5 μM Pro-survival compound (PSC, Merck Millipore, #529659, added only on the day of passaging) and transferred to a new plate coated with Geltrex™. The morphology of iPSCs was checked daily using a Zeiss Axio Vert. A1 microscope.

### 4.2. Cardiomyocyte Generation

The cardiomyocyte differentiation protocol was previously published [17]. In the present project, this protocol was further adapted to each of the cell lines as described in the Results section. Human and rhesus iPSCs were plated on a 12-well plate coated with Geltrex™ and grown in a UPPS medium, which was changed daily for two–three days. To start cardiac differentiation, the medium was changed on day 0 and 24 h later to a Mesodermal induction medium containing RPMI 1640 medium (Thermo Fisher Scientific, #11875093), B27™ (50×) minus insulin (Thermo Fisher Scientific, #A1895601), 200 μM L-Ascorbic acid 2-phosphate (Sigma-Aldrich, #A8960), 1 mM sodium pyruvate (Thermo Fisher Scientific, #11360070), 1 μM Chir99021 (Stemgent, #04–0004), 5 ng/mL BMP4 (PeproTech, Waltham, MA, USA, #120–05) and 9 ng/mL Activin-A (R&D Systems, Minneapolis, MN, USA #338-AC). On days 3 and 5, the medium was replaced by a cardiac induction medium containing RPMI 1640, B27™ (50×) minus insulin, 1 mM sodium pyruvate (Thermo Fisher Scientific, #11360070), 200 μM L-ascorbic acid 2-phosphate (Sigma-Aldrich, #A8960) and 5 μM IWR-1 (Sigma-Aldrich, #I0161). On day 7, the medium was replaced by a cardiomyocyte cultivation medium containing RPMI 1640, B27™ (50×) with insulin and 200 μM L-ascorbic acid 2-phosphate. Cardiomyocytes were further propagated in this medium until day 12 and changed every 2–3 days. Cells were monitored daily, and from day 5, the contractile activity was assessed using bright field microscopy. The first visible spontaneous cell contractions (“beating”) usually occurred around day 6–9, depending on the cell lines. Afterward, the cells were split and used for the metabolic selection phase [17] or fixed for flow cytometry analysis.

Passaging and Pelleting: TrypLE™ Express Enzyme (1X; Gibco, #12604013) was used to passage cardiomyocytes. The cells were washed with 1 mL/well DPBS (Thermo Fisher Scientific, #14190144) and incubated with the enzyme for 10–20 min at 37 °C. Then, cardiomyocytes were detached and transferred to a tube with twice the volume of the cardiomyocyte cultivation media supplemented with 10 µM PSC to inactivate the dissociation reagent. Next, cells were centrifuged at 300× *g* for 5–10 min. The supernatant was discarded, and the cells were plated on new Geltrex™-coated 6-well plates or were used for direct lysis or cell pelleting and frozen at −80 °C for further analysis.

Selection: Cells were split on day 12 using TrypLE™ Express Enzyme (1X), as described above. The next day after splitting, the medium was changed, and the cells were propagated to form a monolayer (2–3 days). Then, the medium was replaced by a selection medium containing RPMI 1640 without glucose (Thermo Fisher Scientific, #11879020), 4 mM lactate (Sigma-Aldrich, #L4263)/HEPES (Roth, #HN78.1) solution, 0.2 mg/mL L-ascorbic acid 2-phosphate and 0.5 mg/mL recombinant human albumin (Sigma-Aldrich, #A9731). The medium was changed every 2–3 days for a week. On day 7, the medium was replaced by the cardiomyocyte cultivation medium, and the cells were left to recover for the next 3–7 days. Following that, cells were split using TrypLE™ Express Enzyme (1X), as described above, between the new plates and used for downstream applications.

### 4.3. Fluorescence-Activated Cell Sorting (FACS)

To perform an FACS analysis, cardiomyocytes were digested with prewarmed TrypLE™ Express Enzyme (1X) for 10–20 min at 37 °C. The dissociation reaction was inactivated with twice the volume of the cardiomyocyte cultivation medium. Afterward, cells were detached by pipetting and centrifuged at 300× *g*, 5–10 min, RT. DPBS containing 4% paraformaldehyde (Merck Millipore, Darmstadt, Germany #104005) was used for fixation for 20–30 min, after which the cells were centrifuged again. Next, the samples were washed with DPBS and centrifuged. The supernatant was discarded and the cells were resuspended in 0.1% Triton X-100 (MP Biomedicals, Santa Ana, CA #02300221) and in 1% BSA (Thermo Fisher Scientific, #15260037)/DPBS for permeabilization and blocking at +4 °C overnight.

On the day of sorting, the pretreated cells were washed with DPBS and centrifuged. After removing DPBS, the cells were resuspended in the respective antibody solutions, diluted with 1% BSA/DPBS and incubated at 37 °C for 1 h. The antibodies used were FITC-conjugated cardiac troponin T (cTNT) (Miltenyi Biotec, #130–119-575, 1:50) and IgG Control REA400 (Miltenyi Biotec, #130–104-611, 1:50). Next, the cells were washed once with DPBS and centrifuged at 300× *g* for 5 min. Eventually, the cells were resuspended in 200–400 μL/tube FACS buffer containing 1% BSA/DPBS and 2 mM EDTA and transferred to a sorting tube using 100 um cell strainers (pluriStrainer^®®^ 100 µm, Pluriselect, Leipzig, Germany).

The cells were then scanned with a Sony SH800S sorter using a gating strategy via dot plot. Each analysis contained unstained, non-specific IgG-incubated and cTNT-FITC-stained samples. Unstained and IgG-incubated samples were used as negative controls and to identify the non-cardiac population. The data were exported using CVS files and analyzed by GraphPad Prism 8 software (Version 8.0.1).

### 4.4. Immunofluorescence Staining

The cells were seeded on glass coverslips (Mensel-Gläser, Braunschweig, Germany #CB00200RA1) coated with Geltrex™ in 6-well plates. Cardiomyocytes were generally seeded at 300,000 cells/well to detect cardiac markers. Human and rhesus iPSCs were seeded at 200,000 cells/well to detect pluripotency markers. The cardiac markers were visualized using cardiac troponin T (Thermo Fisher Scientific, MA5–12960, 1:200), connexin43 (Cx43, Abcam, Cambridge, USA #ab11370, 1:1000), myosin light chain 2a (MLC2a, Synaptic systems, Göttingen, Germany, 311 011, 1:200), myosin light chain 2v (MLC2v, Proteintech, Rosemont, IL, USA #10906–1-AP, 1:200) and α-actinin (Sigma-Aldrich, #A7811. 1:1000) antibodies diluted in 1% BSA/DPBS. Pluripotency factors were visualized using LIN28A (cell signaling, Danvers, MA, USA #3978S, 1:800), NANOG (cell signaling, #4903, 1:400), OCT4A (Abcam, #ab19857, 1:400) and SALL4 (Sigma, HPA015791, 1:400) antibodies diluted in 1% BSA/DPBS.

Initially, the cells were washed once with DPBS and fixed with 4% paraformaldehyde in DPBS for 20 min. Next, the cells were washed three times with DPBS and permeabilized using 0.1% Triton X-100 in 1%BSA/DPBS. After that, the cells were washed three times with DPBS and blocked with 1% BSA in DPBS at +4 °C overnight or longer.

Following blocking, the cells were washed three times with DPBS and incubated with 100 μL/coverslip of primary antibodies at 37 °C for 1 h or at +4 °C overnight and washed thrice with DPBS the next day. Then, the cells were incubated with 100 μL/coverslip secondary antibodies at 37 °C for 1 h and washed three times with DPBS again. The secondary antibodies used were Alexa Fluor 488 donkey-α-rabbit IgG (Thermo Fisher Scientific, #A21206), Alexa Fluor 488 goat-α-mouse IgG (A28175), Alexa Fluor 555 donkey-α-rabbit IgG (A31572) and Alexa Fluor 555 goat-α-mouse IgG (Thermo Fisher Scientific, #A21422) in 1:500 dilution in 1%BSA/DPBS. Next, the nuclei were stained with 1 mL/coverslip of 0.1–0.2 μg/mL DAPI (Sigma-Aldrich, #D9542) diluted in H_2_O for 5 min in the dark. Residues of DAPI were washed three times with DPBS and once with H_2_O. Coverslips were then mounted on slides using 40 μL/slide Fluoromount-G™ (Thermo Fisher Scientific, #00–4958-02). The slides were sealed with nail polish and stored at +4 °C in the dark.

Immunostainings were imaged using an epifluorescence microscope Zeiss Observer Z1 and analyzed using ImageJ software (Version 1.54f).

### 4.5. Proliferation Analysis

An EdU Click-555 Roti^®®^ Imaging kit (Roth, Karlsruhe, Germany #1Y72.1) was used for the detection of proliferating iPSCs. Human iPSCs were seeded as 200,000 cells/well, and rhesus iPSCs were seeded on Geltrex-coated glass coverslips on 6-well plates. The next day, the cells were exposed to EdU for two hours and processed according to the manufacturer’s instructions. The nuclei were stained with DAPI. Coverslips were mounted on slides using 40 μL/slide Fluoromount-G™. The mounting was sealed with nail polish, and the slides were stored at +4 °C in the dark. Immunostainings were imaged using a Zeiss Observer Z1, and the results were analyzed via ImageJ software using manual nuclei counting.

## 5. Conclusions

We conclude that even if iPSC lines are generated according to the same protocol in the same laboratory, their characteristics and behavior in experimental procedures can vary greatly. Even the extensive standardization of procedures results in heterogeneous iPSC lines, as we have shown in this study for optimized cell seeding densities in the course of myocardial differentiation from iPSCs. These results show that no generalized conclusions should be drawn on the basis of only one or two iPSC lines. 

## Figures and Tables

**Table 1:** Overview of the cell lines of human and rhesus macaque origin.

Cell Line	Name	Species	Sex	Source
hiPSC1	iLonza2.2	*Homo* *sapiens*	Male	Foreskin fibroblasts (purchased from Lonza)
hiPSC2	iLonza2.4	*Homo* *sapiens*	Female	Skin fibroblasts (purchased from Lonza)
RhiPSC1	iRh33.1	*Macaca mulatta*	Male	Skin fibroblasts
RhiPSC2	iRh24.4	*Macaca mulatta*	Female	Skin fibroblasts

## Data Availability

The additional data can be provided by the authors upon reasonable request.

## Supplementary Materials

The following supporting information can be downloaded at: https://www.mdpi.com/article/10.3390/ijms25158449/s1.
