# Supplementary material for: The Efficiency of In Vitro Differentiation of Primate iPSCs into Cardiomyocytes Depending on Their Cell Seeding Density and Cell Line Specificity
Source: Int J Mol Sci. 2024 Aug 2;25(15):8449. doi: 10.3390/ijms25158449 (PMC11312487; doi:10.3390/ijms25158449)
Supplement: Supplementary file 1 [file ijms-25-08449-s001.zip › ijms-3038209-supplementary.pdf]

Table S1. Descriptive statistics of values (%) used for proliferation analysis of iPSCs.

	hiPSC1	hiPSC2	RhiPSC1	RhiPSC2
**Number of values**	30	30	30	28
**Minimum**	39,68	43,33	36,84	42,31
**Maximum**	81,93	89,58	92,31	85,42
**Range**	42,25	46,25	55,47	43,11
**Mean**	63,48	62,08	66,94	62,60
**Std. Deviation**	11,76	12,33	15,84	12,28
**Std. Error of Mean**	2,148	2,251	2,891	2,321

Table S2. Percentage of cTNT-positive cells of hiPSC1 cell line after differentiation before selection. Four independent experiments were used for each group of cell seeding numbers.

Number of seeded cells/well	#1 (%)	#2 (%)	#3 (%)	#4(%)
**150,000**	45,43	44,42	37,22	40,32
**200,000**	50,25	52,93	60,82	50,21
**250,000**	59,26	50,73	63,56	56,56
**300,000**	75,89	79,02	70,46	79,39
**350,000**	71,06	66,28	73,99	69,16

Table S3. Percentage of cTNT-positive cells of hiPSC2 cell line after differentiation before selection. Three to four independent experiments were used for each group of cell seeding numbers.

Number of seeded cells/well	#1 (%)	#2 (%)	#3 (%)	#4(%)
**40,000**	23,27	26,99	30,88	26,8
**50,000**	31,8	30,54	36,73	
**60,000**	69,56	79,45	81,7	
**70,000**	75,48	79,07	74,51	77,03
**80,000**	73,72	78,32	75,12	
**90,000**	72,54	70,06	75,65	67,82

Table S4. Percentage of cTNT-positive cells of RhiPSC1 cell line after differentiation before selection. Three to four independent experiments were used for each group of cell seeding numbers.

Number of seeded cells/well	#1 (%)	#2 (%)	#3 (%)	#4(%)
**80,000**	44,56	37,71	33,54	44,04
**100,000**	42,74	50,57	56,5	36,13
**120,000**	60,69	56,96	62,9	51,23
**140,000**	52,68	51,24	65,88	55,38
**160,000**	20,28	11,65	38,4	
**180,000**	25,75	13,04	23,45	

Table S5. Percentage of cTNT-positive cells of RhiPSC2 cell line after differentiation before selection. Three to four independent experiments were used for each group of cell seeding numbers.

Number of seeded cells/well	#1 (%)	#2 (%)	#3 (%)	#4(%)
**70,000**	35,74	40,4	39,6	30,1
**90,000**	61,89	65,86	66,17	65,37
**110,000**	27,46	17,21	27,46	
**130,000**	4,89	5,48	8,11	
**150,000**	13,2	16,8	2,51	

Table S6. Percentage of cTNT-positive cells in hiPSC1, hiPSC2, RhiPSC1, and RhiPSC2 after metabolic selection. Three independent experiments were used for each group.

Cell line	#1 (%)	#2 (%)	#3 (%)
**hiPSC1**	97,65	97,04	97,72
**hiPSC2**	74,66	72,09	70,03
**RhiPSC1**	82,44	86,16	92,99
**RhiPSC2**	95,71	95,21	96,13

Figure S1

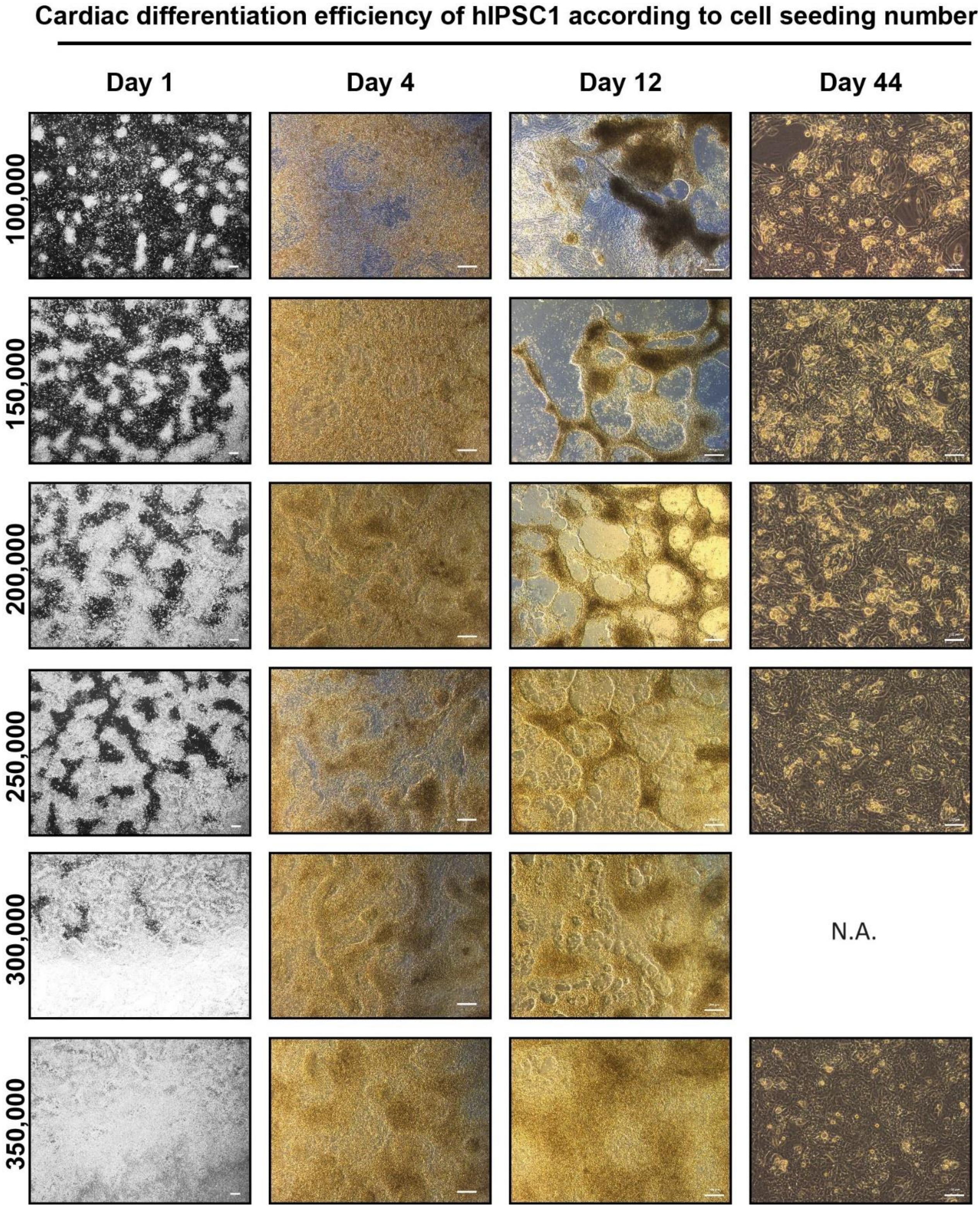

Figure S1: Overview of the morphology of the hiPSC1 line at day 1, 4, 12 and 44 of differentiation into cardiomyocytes depending on the cell seeding density. Scale bars represent X μm. N.A., no image available.

Figure S2

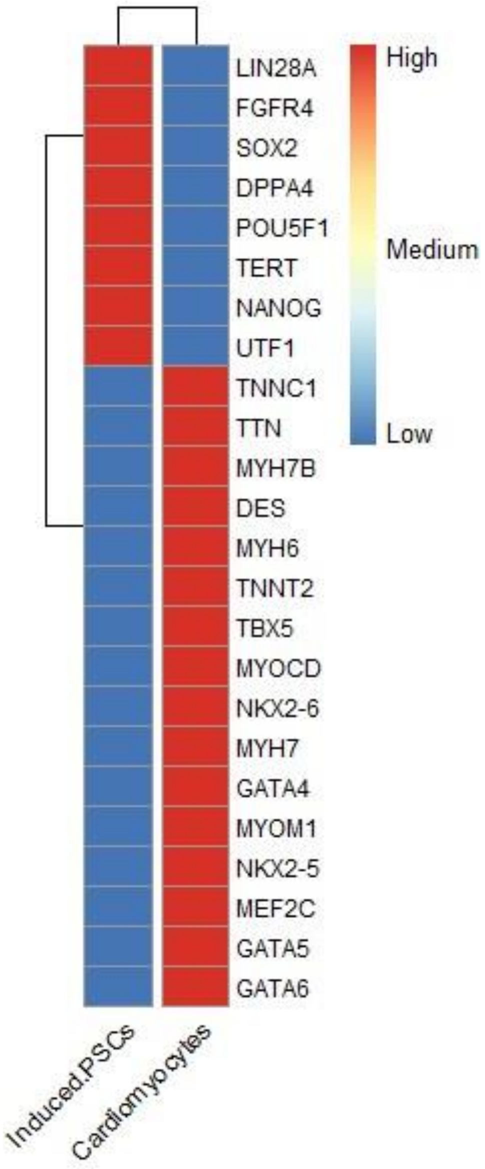

Figure S2: Expression of a selected set of pluripotent stem cell and cardiomyocyte markers in hiPSC1 and in cardiomyocytes derived from hiPSC1.
